# Psychostimulant Drugs and Neuroplasticity

**DOI:** 10.3390/ph4070976

**Published:** 2011-06-30

**Authors:** Emilio Fernandez-Espejo, Nieves Rodriguez-Espinosa

**Affiliations:** Departamento de Fisiología Médica, Facultad de Medicina, Universidad de Sevilla, Av. Sanchez Pizjuan 4, 41009 Sevilla, Spain

**Keywords:** cocaine, amphetamine, neuroplasticity

## Abstract

Drugs of abuse induce plastic changes in the brain that seem to underlie addictive phenomena. These plastic changes can be structural (morphological) or synaptic (biochemical), and most of them take place in the mesolimbic and mesostriatal circuits. Several addiction-related changes in brain circuits (hypofrontality, sensitization, tolerance) as well as the outcome of treatment have been visualized in addicts to psychostimulants using neuroimaging techniques. Repeated exposure to psychostimulants induces morphological changes such as increase in the number of dendritic spines, changes in the morphology of dendritic spines, and altered cellular coupling through new gap junctions. Repeated exposure to psychostimulants also induces various synaptic adaptations, many of them related to sensitization and neuroplastic processes, that include up- or down-regulation of D1, D2 and D3 dopamine receptors, changes in subunits of G proteins, increased adenylyl cyclase activity, cyclic AMP and protein kinase A in the nucleus accumbens, increased tyrosine hydroxylase enzyme activity, increased calmodulin and activated CaMKII in the ventral tegmental area, and increased deltaFosB, c-Fos and AP-1 binding proteins. Most of these changes are transient, suggesting that more lasting plastic brain adaptations should take place. In this context, protein synthesis inhibitors block the development of sensitization to cocaine, indicating that rearrangement of neural networks must develop for the long-lasting plasticity required for addiction to occur. Self-administration studies indicate the importance of glutamate neurotransmission in neuroplastic changes underlying transition from use to abuse. Finally, plastic changes in the addicted brain are enhanced and aggravated by neuroinflammation and neurotrophic disbalance after repeated psychostimulants.

## 1. Introduction

The term neuronal plasticity, in wide sense, refers to the neurobiological basis that allows adaptive changes in behavior, motivation and emotions, and it is also on the basis of maladaptive changes related to addictive phenomena [1]. Two types of neuroplasticity can be distinguished: structural plasticity and synaptic plasticity. Structural plasticity refers to changes in neuronal morphology (including axons, dendrites and dendritic spines), the genesis and suppression of synapses, and the genesis of new neurons (neurogenesis) and neurites (neuritogenesis). Synaptic plasticity refers to biochemical and neurochemical modifications related to changes in synaptic activity, which leads to enduring changes in synaptic efficacy, and in behavior accordingly. Other addiction-related processes are associated with neuroplasticity, for instance drug sensitization, self-administration, drug-seeking and hypofrontality. These phenomena have been analyzed in depth, mostly in relation to repeated psychostimulants, during the last years.

Addictive drugs of abuse have the potential to induce addiction through enduring changes in neuronal plasticity, altering neurotransmission systems, the strength and duration of synaptic contacts, neuronal morphology, etc. In other terms, addiction is a disease whose neuropathology is based on aberrant structural and synaptic plasticity. By changing brain circuits, addictive drugs impair the development of behavioral strategies towards biological stimuli in favor of progressively greater orientation of behaviors towards drug seeking and drug-taking strategies. Enduring drug-induced neuroplasticity establishes a maladaptive orientation to the environment that manifests as: i) impaired ability to regulate the drive to obtain and use drugs, and ii) reduced drive to obtain natural rewards. Psychostimulant drugs such as amphetamine and cocaine are prototypic drugs inducing neuroplasticity changes, and they have been extensively studies during the last years.

## 2. Sensitization, a Model of Neural Plasticity

The repeated administration of drugs of abuse such as cocaine and amphetamine produces a progressive increase in locomotor activity over time, referred as motor sensitization. Motor sensitization is considered as a model of neural plasticity [2-4], and neuroadaptations mediating sensitization could underlie some of the behavioral changes associated with chronic drug abuse [5]. Chronic psychostimulant exposure produces biochemical adaptations in specific brain regions thought to mediate activating properties of these drugs [3,6]. The mesolimbic system is the most important system involved in these adaptations, which comprises dopaminergic neurons in the ventral tegmental area (VTA) and their projections to the limbic forebrain such as the nucleus accumbens [3,7]. Other structures such as prefrontal cortex, amygdala and hippocampus are also involved. Recently Perez *et al.* [8] reported that sensitization to cocaine generates a high efficiency of hippocampal synaptic plasticity that may underlie the aberrant engagement of learning processes occurred during drug addiction.

At a biochemical level, tyrosine hydroxylase (TH) and N-methyl-D-aspartate receptor 1 (NMDAR1) expression in the VTA are found to be increased after repeated psychostimulant and other drugs of abuse [9-12], and these changes are known to be related to motor sensitization. Thus TH up-regulation leads to augmented dopamine neurotransmission in the VTA and nucleus accumbens [9,10], a critical feature for the development of sensitization. Repeated drug exposure is known to increase extracellular dopamine levels in the nucleus accumbens [13,14], and acute or chronic cocaine directly induces augmentation of dopamine release in the nucleus accumbens. Repeated cocaine exposure also leads to down-regulation of D2 receptors in the ventral tegmental area, together with sensitization of D1 receptors located to the glutamatergic terminals of the VTA, all together inducing an augmented dopaminergic activity in this area as well [3,15-18]. NMDA receptors become more permeable to calcium after chronic cocaine's administration, another critical feature in the motor sensitization process [19]. D1 receptors of the nucleus accumbens become upregulated during sensitization, and infusion of a D1R agonist into this nucleus are known to enhance cocaine-induced behavioural sensitization [20]. NMDARs are located on non-DA neurons within the VTA and they play a major role in cocaine-induced sensitization addictive behavior [21].

The endocannabinoid system is also involved in sensitization processes to psychostimulants. The brain contains an endogenous cannabinoid system that participates in several processes such as nociception, emotion, motor control, and reward [22]. The endocannabinoid system and CB_1_ receptors (CB_1_Rs) are involved not only in the reinforcement properties of Δ^9^-tetrahydrocannabinol [23], but also of several drugs of abuse such as cocaine [24]. The overall picture is that cannabinoid CB_1_R appear to be involved in the persistence of cocaine addiction [25], affecting the expression, not induction, of sensitization. Thus, systemic injections of rimonabant, ligand of CB_1_ receptors with rimonabant does not affect the development of cocaine's sensitization in rats [26], but the expression of cocaine's sensitization has been reported to be reduced by pretreatment with rimonabant [27,28]. Silencing accumbal CB_1_R with siRNAs (using lentiviral-mediated silencing of CB_1_R) leads to strong increase in motor activating effects of cocaine, and motor sensitization is not affected (unpublished results).

Our studies indicate that, at the level of the ventral tegmental area, D-serine, an endogenous ligand of the glutamatergic NMDA receptor, is involved in sensitization effects of psychostimulants [29]. Astrocytes and neurons express D-amino acid oxidase (DAO, synthesizing enzyme for D-serine) and release D-serine [30-32]. D-serine modulates NMDA receptor activity in the central nervous system [33-35], and it acts through binding to NMDA receptors at the glycine site, thereby facilitating their activation [25]. In this context, sensitization-related changes in the VTA have been compared to long-term potentiation (LTP) in the hippocampus. Besides it is known that a single injection of cocaine can induce LTP of AMPA receptor-mediated current in dopaminergic neurons in the VTA [36]. Stimulation of NMDA receptors is needed for the development of cocaine's sensitization [37-39]. The NMDA receptor consists of several distinct binding sites identified by the ligand: glutamate or NMDA, PCP or MK-801, glycine, as well as Mg^2+^-binding site. D-Serine and glycine are co-agonists with glutamate for the NMDA receptor ionophore [40], and co-agonism in the VTA is needed for the initiation of locomotor sensitization to cocaine.

Sensitization is also mediated by calcium and calmodulin-kinase II (CaMKII) signaling in the ventral tegmental area. Thus, enhanced levels of calcium in the VTA mediate locomotor activating effects of cocaine [16,18]. Glutamatergic AMPA and NMDA receptors along with L-type calcium channels are involved in these activating effects, because they become more permeable to calcium after cocaine's administration [18]. Calcium binds calmodulin (calcium/CaM complex) and then calcium-dependent kinases such as CaMKII bind calcium/CaM become phosphorylated and activated by calmodulin kinase-kinase (CaMKK). It is well known that cocaine injections enhance total CaMKII protein in the VTA [39]. Suppressing CaMKII activity in the ventral tegmental area enhances the acute response to 15 mg/kg cocaine, and attenuates the initiation of cocaine-induced behavioral sensitization in rats [39]. CaMKII is a transducer of calcium signaling, this kinase being expressed in many brain regions [41,42], including the VTA [39]. Calcium signaling pathways play a pivotal role in synaptic plasticity and memory formation [43,44], as well as CaMKII [45]. CaMKII functions as a potent stimulator of calcium-dependent gene expression, and it activates several transcription factors such as CREB, which is phosphorylated on the regulatory Ser133 residue [46,47]. CREB activation in the VTA is directly involved in sensitization as explained, and regulates tyrosine hydroxylase (TH) transcription [48].

## 3. Structural Plasticity after Psychostimulant Consumption

Repeated exposure to psychostimulants induces morphological changes in the mesolimbic target areas such as increase in the number of dendritic spines, changes in the morphology of dendritic spines, and altered cellular coupling through new gap junctions [49-51]. Several experimental data suggest that rearrangement of neural networks must develop for the long-lasting plasticity required for sensitization to occur. Protein synthesis inhibitors block the development of sensitization to cocaine [52], and pups younger than three weeks old, which are too immature to develop neural networks, do not become sensitized to psychostimulants [53]. Hence sensitization is also linked to morphological changes in rewarding circuits.

Arc mRNA and Arc protein, markers for neuritic outgrowth, have been reported to be upregulated in the dorsal striatum after chronic cocaine in rats [54], and downregulation of mielin-related genes takes place in the nucleus accumbens of human cocaine abusers [55]. Enhancement of deltaFosB and AP-1 proteins has been postulated to subserve these morphological changes [3]. In fact, the transcription factor deltafosB accumulates in dopamine-terminal fields in the cortex and striatum [3,56], and it is a strong candidate for mediating enduring changes in these regions. In fact, increase in dendritic spine density has been reported in accumbens spiny neurons during extended abstinence from psychostimulants [57], and this increase is mediated by deltaFosB stimulation of Cdk5 [51].

Another change in protein synthesis that is particularly important in establishing drug-induced neuroplasticity is a rise in brain-derived neurotrophic factor (BDNF). The enduring changes in BDNF accumulate with increasing period of abstinence [27,58,59], and BDNF promotes forms of synaptic plasticity such as dendritic spine formation [60]. This protein is another stable neuroplasticity candidate that may contribute to persistence of addiction and drug-seeking behavior.

It is of great interest that similar neuroplastic changes take place in different reinforcement-related behaviors. Thus repeated sexual behavior induces a sensitized locomotor response to amphetamine in rats, and the number of dendrites and spines in the nucleus accumbens is increased with sexual experience. Sexual experience induces functional and morphological alterations in the mesolimbic system similar to repeated exposure to psychostimulants, hence some alterations in the mesolimbic system are common for natural and drug reward and might play a role in general reinforcement [61].

## 4. Self-Administration and Neuroplasticity

There is an important literature on the neuroplastic changes at excitatory synapses in the brain reward circuits associated with psychostimulant self-administration in rodent, an experimental model which is much more likely to help understanding the neural basis of drug addiction. In fact, several studies have demonstrated that voluntary drug intake through a self-administration paradigm induce different changes to those observed after non-contingent drug-exposure [62-65]. Self-administration studies indicate that cocaine induces an enhancement of glutamatergic function in VTA dopamine neurons. This effect of cocaine is not by itself because it is necessary a strong pairing between the drug and a cue, pointing to the great importance of conditioned responses for synaptic plasticity after drugs of abuse [65]. Glutamate receptors such as mGlu5 receptors are involved in cocaine-induced plasticity in VTA dopaminergic cells, but mGlu5 receptor may not be essential for psychostimulant behavioural sensitization and consolidation of addiction [66].

In this context, the nucleus accumbens and its NMDA receptors, another type of glutamate receptors, are more involved in persistent addiction, through the modulation of long term depression (LTD), a form of synaptic plasticity. Kasanetz *et al.* [67] have suggested that a failure of an individual to counteract the impairment in NMDAR-LTD after chronic cocaine contributes to the transition to addiction. Self-administration studies also indicate that AMPA receptors in the nucleus accumbens are involved in cocaine-seeking after addiction consolidation. Thus the number of AMPA receptors in the nucleus accumbens is increased after prolonged abstinence from cocaine self-administration. New AMPA receptors lacking glutamate receptor 2 (GluR2) are added, and these receptors mediate the incubation of drug craving [68]. All these studies strengthen the concept than transition from use to abuse takes place in limbic structures such as the nucleus accumbens and that glutamate neurotransmission is associated to plastic changes mediating this transition and craving responses during withdrawal.

## 5. Neuroimaging and Psychostimulants

Addiction-related changes in cortical circuits has been visualized in addicts to psychostimulants using neuroimaging techniques. These studies indicate that there is a reduction in prefrontal cortex (PFC) metabolism and blood flow in addicted to cocaine [69]. This “hypofrontality” is a strong indicator of reduced ability to regulate drug-seeking or to inhibit motivational drives related to drug addiction, because affected regions are anterior cingulate and ventral orbital cortex, which are strongly linked to motivated behavior, switching and attention responses [70,71]. D2 dopamine receptor functionality seems to be linked to this hypofrontality, as suggested by Volkow *et al* [72]. These authors observed that there is an association between level of dopamine D2 receptors, and metabolism in the orbitofrontal cortex in methamphetamine and cocaine abusers. They propose that D2 receptor-mediated dysregulation of the orbitofrontal cortex could underlie a common mechanism for loss of control and compulsive drug intake in drug-addicted subjects. Furthermore, if addicted subjects are exposed to cues previously associated to the drug, there is a marked activation in the prefrontal cortex [69,73]. The activity in the PFC has been positively correlated with the intensity of cue-induced desire for the drug, an index for craving.

Psychostimulants induce dopamine release in ventral and dorsal striatal nuclei. Thus Willeit *et al.* [74] have observed that D-amphetamine induces displacement of the D2/3 agonist radioligand [^11^C]-(+)-4-propyl-9-hydroxynaphthoxazine [(+)-PHNO], a neuroimaging way to measure dopamine release in the living human brain. Sensitization to amphetamine has also been modeled using neuroimaging by Boileau *et al* [75]. Thus they report that an initial dose of amphetamine causes dopamine release in the ventral striatum, as evaluated by the reduction in [^11^C]raclopride binding. Consistent with a sensitization-like phenomenon, 14 and 365 days after the third dose of amphetamine there is a greater psychomotor response and increased dopamine release (a greater reduction in [^11^C]raclopride binding), relative to the initial dose, in the ventral striatum, progressively extending to the dorsal caudate and putamen. This phenomenon persists for at least one year.

Neuroimaging techniques have also revealed that there is a reduction in dopamine receptor activation in response to low doses of psychostimulants [76,77]. Methylphenidate-induced dopamine release into the striatum is impaired in cocaine addicts, and addicts show reduced levels of D2 receptors in the striatum [78]. Subjects with low D2 density report pleasurable effects from methylphenidate, whereas subjects with higher D2 density do not like the effects of this psychostimulant drug [79]. The binding of cocaine and other psychostimulants to DAT can be assessed in humans by measuring the dose-dependent displacement of [^11^C]cocaine by a non-radiactive competitor. The extent of euphoria experienced following administration of cocaine correlated positively with the occupancy of [^11^C]cocaine-binding sites in the striatum, and all subjects with greater than 60% occupancy of DAT experienced euphoria [80].

Neuroimaging studies confirm that cocaine and methamphetamine abuse induce down-regulation of D2 receptors, and this downregulation wears off after prolonged abstinence. Thus in chronic cocaine users, [^11^C]raclopride is reduced by 15% in the striatum [81], and simple ratio of [^11^C]NMSP uptake in the striatum is also reduced, but returns to normal after prolonged abstinence [82]. Regarding methamphetamine abusers, it has been detected a 10-15% reduction in striatal [^11^C]raclopride binding [72].

Finally, treatment response in cocaine abusers is related to dopamine signaling as recently demonstrated by Martinez *et al*. [83], who have reported that low dopamine transmission is associated with treatment failure. Thus they quantified, prior to treatment, [^11^C]raclopride binding before and after the administration of methylphenidate, and the outcome measures were lower in the volunteers who did not respond to treatment than in those who experienced a positive treatment response. The authors propose that the combination of behavioral treatment with methods that increase striatal dopamine signaling might serve as a therapeutic strategy for cocaine dependence [83].

## 6. Psychostimulants and Neuroinflammation

Neuroinflammation of the central nervous system seems to participate in neuroplasticity changes and sensitizing effects of drugs such as psychostimulants [84-86]. It worth recalling that inflammation is part of a complex biological response to harmful stimuli, such as pathogens or irritants. Drugs of abuse could be included among irritants, and neuroinflammation would be a protective attempt by the organism to remove the injurious stimuli. In this context, it is well known that psychostimulants enhance activation of inflammatory cells such as astrocytes and glial cells in neocortex [87]. Activated astrocytes and glia release numerous pro-inflammatory factors such as nitric oxide, ATP, TGF-α, interleukin-1β, interleukin 2 and interleukin 6 [88-90], and several inflammatory cytokines such as interferon-gamma and interleukin 10 are enhanced by chronic cocaine [91]. Cocaine is also known to enhance the transcription factor nuclear factor-kappaB (NF-κB), and it reduces levels of TNF-α [92]. In this context, NF-κB is an important inflammatory factor that controls many genes involved in inflammation, and NF-κB is found to be chronically active in many inflammatory diseases.

Apart from proinflammatory factors released by astrocytes and glia, it is of interest that peroxisome proliferator-activated receptors (PPARs) are involved in neuroinflammation and drug dependence, because PPAR-γ has a significant role in the expression of motor sensitization to metamphetamine in mice [86]. Peroxisome proliferator-activated receptor is a ligand-activated transcription factor belonging to a nuclear hormone receptor superfamily. Three PPAR isotypes (α, β, γ) have been characterised and can be translocated to the nuclear fraction of cells as heterodimers with the retinoid X receptor (RXR). The ligand-activated heterodimers can bind to PPAR response element (PPRE) to regulate the transcription of target genes. Inflammation is known to be regulated by PPAR-γ and PPAR-α, whose activation negatively regulates the transcription of inflammatory response genes by antagonizing the AP-1 and NF-κB signalling pathways [93-95]. In fact, PPARs play prominent roles in several physiological processes including the inflammatory response apart from their role in the control of lipid and lipoprotein metabolism, and glucose homeostasis [96-98]. Recently we have observed that PPAR-α plays a homeostatic role attenuating motor sensitizing effects of repeated morphine treatment, but not cocaine, likely through a reduction of inflammation-associated changes [99]. It seems that, among PPARs, PPAR-γ but not PPAR-α, is more involved in sensitizing effects of psychostimulants. However it is worthnoting that PPAR-α has been linked to the reinforcing effects of some drugs such as nicotine, because PPAR-α agonists supress nicotine-induced activation of mesolimbic dopamine neurons through PPAR-α [100].

## 7. Psychostimulants and Altered Neurotrophism

The emergence of neurotrophic disbalance, mainly within the mesolimbic regions and other regions such as amygdala and hippocampus, seems to be important in the development of addictive processes and neuroplastic changes after psychostimulants and other drugs of abuse. Neurotrophic factors have been implicated in many forms of plasticity in the adult nervous system, hence changes in dopamine systems after repeated drugs suggest that neurotrophic factors such as GDNF, BDNF or FGFs are involved in drug's addictive processes [3,9,101-106].

Among these trophic factors, fibroblast growth factors (FGFs) and their receptors participate in many processes such as regulation of synaptic plasticity, learning and memory [107], and FGF-2 (fibroblast growth factor-2) has been involved in the sensitizing effects of psychostimulants [105,106]. Fibroblast growth factor-1 or FGF-1, another member of the FGFs family, is also involved in the regulation of synaptic plasticity [107], and it is implicated in sensitization to drugs of abuse [108]. Fumagalli *et al.* [109] have demonstrated that stress interacts with cocaine to alter the pattern of FGF-2 expression in a regionally selective fashion. These results identify a potential molecular target through which stress alters cellular sensitivity to cocaine and might prove useful in understanding the mechanisms underlying brain vulnerability to stress.

At the level of ventral tegmental area, deficit of GDNF and upregulation of neurotrophins such as NT-3 are linked to sensitization phenomena after psychostimulants. This dual change represents quite well the emergence of a neurotrophic disbalance during addiction, because both GDNF dowregulation and NT-3 upregulation facilitates dopamine activity within the VTA. GDNF is released by dopamine neurons of VTA acting retrogradely on GABAergic terminals enhancing GABA release after enhancement of the calcium binding protein frequenin inside terminals [110]. Downregulation of GDNF leads to a higher activity of dopamine neurons that as a consequence are subjected to less inhibition from GABAergic interneurons.

As already mentioned, another change in protein synthesis that is particularly important in establishing drug-induced neuroplasticity is a rise in brain-derived neurotrophic factor (BDNF). Changes in levels of BDNF protein and mRNA have been observed in the brain following administration of drugs of abuse such as psychostimulants, and these changes have been observed in the nucleus accumbens, prefrontal cortex, ventral tegmental area, and amygdala [111-114]. BDNF signaling can induce different effects depending on the brain region examined. For instance, increased BDNF in the NAc enhances cocaine-induced behaviors [113], whereas in the prefrontal cortex BDNF supresses them [115]. Besides BDNF infused into the hippocampus is antidepressant, but in the nucleus accumbens exerts prodepressant effects. In the medial prefrontal cortex, upregulated BDNF facilitates LTP and contributes to neurobehavioral adaptations to psychostimulants [116]. Cortico-tegmental BDNF is also involved in long-term amphetamine sensitization [117].

Calcium influx and CaMKII have been also implicated in the production and responsiveness to BDNF [118,119]. BDNF participates on the development of sensitization to cocaine or amphetamine because direct infusion of BDNF into the VTA blocks the ability of cocaine to produce some biochemical and morphological effects in this region, such as the induction of tyrosine-hydroxylase [9,101], and repeated amphetamine treatment increases BDNF within the VTA, an effect that is enhanced in sensitized animals [117]. BDNF also participates during abstinence, and recently it has been observed that elevated BDNF expression after cocaine withdrawal sensitizes the excitatory synapses in the medial prefrontal cortex, and this fact may contribute to cue-induced drug craving and drug-seeking behavior [116]. In the medial prefrontal cortex, upregulated BDNF facilitates LTP and contributes to neurobehavioral adaptations to psychostimulants.

## 8. Conclusions

Psychostimulants induce plastic changes in the brain that seem to underlie addictive phenomena. These plastic changes take place in the mesolimbic and mesostriatal circuits. Several addiction-related changes in brain circuits (hypofrontality, sensitization, morphological alterations) as well as the outcome of treatment have been visualized in addicts to psychostimulants using neuroimaging techniques. Repeated exposure to psychostimulants induces morphological changes such as increase in the number of dendritic spines or changes in the morphology of dendritic spines. Besides repeated exposure to psychostimulants also induces various synaptic adaptations, many of them related to sensitization and neuroplastic processes, that include up- or down-regulation of D1, D2 and D3 dopamine receptors, changes in subunits of G proteins, increased adenylyl cyclase activity, cyclic AMP and protein kinase A in the nucleus accumbens, increased tyrosine hydroxylase enzyme activity, increased calmodulin and activated CaMKII in the ventral tegmental area, and increased deltaFosB, c-Fos and AP-1 binding proteins. Most of these changes are transient, suggesting that more lasting plastic brain adaptations should take place. Self-administration studies indicate the importance of glutamate neurotransmission in neuroplastic changes underlying transition from use to abuse. Finally, plastic changes in the addicted brain are enhanced and aggravated by neuroinflammation and neurotrophic disbalance after chronic psychostimulant administration.

## References

[1] Malenka R.C., Bear M.F. (2004). LTP and LTD: An embarrassment of riches. Neuron.

[2] Wise R.A. (2000). Addiction becomes a brain disease. Neuron.

[3] Nestler E.J. (2001). Molecular basis of long-term plasticity underlying addiction. Nature Rev. Neurosci..

[4] Kelley A.E. (2004). Memory and addiction: Shared neural circuitry and molecular mechanisms. Neuron.

[5] Robinson T.E., Berridge K.C. (2000). The psychology and neurobiology of addiction: An incentive-sensitization view. Addiction.

[6] Koob G.F., Bloom F.E. (1988). Cellular and molecular mechanisms of drug dependence. Science.

[7] Wise R.A., Rompre P.P. (1989). Brain dopamine and reward. Annu. Rev. Psychol..

[8] Perez M.F., Gabach L.A., Almiron R.S., Carlini V.P., de Barioglio S.R., Ramirez O.A. (2010). Different chronic cocaine administration protocols induce changes on dentate gyrus plasticity and hippocampal dependent behavior. Synapse.

[9] Beitner-Johnson D., Nestler E.J. (1991). Morphine and cocaine exert common chronic actions on tyrosine hydroxylase in dopamine brain reward regions. J. Neurochem..

[10] Berhow M.T., Russell D.S., Terwilliger R.Z., Beitner-Johnson D., Self D.W., Lindsay R.M., Nestler E.J. (1995). Influence of neurotrophic factors on morphine- and cocaine-induced biochemical changes in the mesolimbic dopamine system. Neuroscience.

[11] Fitzgerald L.W., Ortiz J., Hamedani A.G., Nestler E.J. (1996). Drugs of abuse and stress increase the expression of GluR1 and NMDAR1 glutamate receptor subunits in the rat ventral tegmental area: Common adaptations among cross-sensitizing agents. J. Neurosci..

[12] Jones S.W. (1998). Overview of voltage-dependent calcium channels. J. Bioenerg. Biomembr..

[13] di Chiara G., Imperato A. (1988). Drugs of abuse preferentially increase synaptic dopamine concentrations in the mesolimbic system of freely moving rats. Proc. Nat. Acad. Sci. USA.

[14] Spanagel R., Almeida O.F.X., Shippenburg T.S. (1993). Long-lasting changes in morphine-induced mesolimbic dopamine release after chronic morphine exposure. Synapse.

[15] Stewart J., Vezina P. (1989). Microinjections of SCH-23390 into the ventral tegmental area and substantia nigra pars reticulate attenuate the development of sensitization to the locomotor activating effects of systemic amphetamine. Brain Res..

[16] Pierce R.C., Born B., Adams M., Kalivas P.W. (1996). Repeated intra-ventral tegmental area administration of SKF-38393 induces behavioral and neurochemical sensitization to a subsequent cocaine challenge. J. Pharmacol. Exp. Ther..

[17] Kalivas P.W., Duffy P. (1998). Repeated cocaine administration alters extracellular glutamate in the ventral tegmental area. J. Neurochem..

[18] Licata S.C., Schmidt H.D., Pierce R.C. (2004). Suppressing calcium/calmodulin-dependent protein kinase II activity in the ventral tegmental area enhances the acute behavioural response to cocaine but attenuates the initiation of cocaine-induced behavioural sensitization in rats. Eur. J. Neurosci..

[19] Licata S.C., Pierce R.C. (2003). The roles of calcium/calmodulin-dependent and Ras/mitogen-activated protein kinases in the development of psychostimulant-induced behavioral sensitization. J. Neurochem..

[20] de Vries T.J., Cools A.R., Shippenberg T.S. (1998). Infusion of a D-1 receptor agonist into the nucleus accumbens enhances cocaine-induced behavioural sensitization. Neuroreport.

[21] Luo Y., Good C.H., Diaz-Ruiz O., Zhang Y., Hoffman A.F., Shan L., Kuang S.Y., Malik N., Chefer V.I., Tomac A.C. (2010). NMDA receptors on non-dopaminergic neurons in the VTA support cocaine sensitization. PLoS One.

[22] Pertwee R.G. (1997). Pharmacology of cannabinoid CB_1_ and CB2 receptors. Pharmacol. Ther..

[23] Maldonado R., Rodriguez de Fonseca F. (2002). Cannabinoid addiction: Behavioral models and neural correlates. J. Neurosci..

[24] de Vries T.J., Shaham Y., Homberg J.R., Crombag H., Schuurman K., Dieben J., Vanderschuren L.J., Schoffelmeer A.N. (2001). A cannabinoid mechanism in relapse to cocaine seeking. Nature Med..

[25] Wiskerke J., Pattij T., Schoffelmeer A.N., de Vries T.J. (2008). The role of CB1 receptors in psychostimulant addiction. Addict. Biol..

[26] Lesscher H.M.B., Hoogveld E., Burbach P.H., van Ree J.M., Gerrits M.A. (2005). Endogenous cannabinoids are not involved in cocaine reinforcement and development of cocaine-induced behavioural sensitization. Eur. Neuropsychopharmacol..

[27] Filip M., Golda A., Zaniewska M., McCreary A.C., Nowak E., Kolasiewicz W., Przegaliński E. (2006). Involvement of cannabinoid CB1 receptors in drug addiction: Effects of rimonabant on behavioral responses induced by cocaine. Pharmacol. Rep..

[28] Gerdeman G.L., Schechter J.B., French E.D. (2008). Context-specific reversal of cocaine sensitization by the CB(1) cannabinoid receptor antagonist rimonabant. Neuropsychopharmacology.

[29] Fernández-Espejo E., Ramiro-Fuentes S., Portavella M., Moreno-Paublete R. (2008). Role for D-serine within the ventral tegmental area in the development of cocaine's sensitization. Neuropsychopharmacology.

[30] Yang Y., Ge W., Chen Y., Zhang Z., Shen W., Wu C., Poo M. (2003). Contribution of astrocytes to hippocampal long-term potentiation through release of D-serine. Proc. Natl. Acad. Sci. USA.

[31] Park K., Shishido Y., Ichise-Shishido S., Kawazoe T., Ono K., Iwana S., Tomita Y., Yorita K., Sakai T., Fukui K. (2006). Potential role for astroglial d-amino acid oxidase in extracellular D-serine metabolism and cytotoxicity. J. Biochem.(Tokyo).

[32] Kartvelishvily E., Shleper M., Balan L., Dumin E., Wolosker H. (2006). Neuron-derived D-serine release provides a novel means to activate N-methyl-D-aspartate receptors. J. Biol. Chem..

[33] Schell M.J., Brady R.O., Molliver M.E., Snyder S.H. (1997). D-serine as a neuromodulator: Regional and developmental localizations in rat brain glia resemble NMDA receptors. J. Neurosci..

[34] Haydon P.G. (2001). Glia: Listening and talking to the synapse. Nat. Rev. Neurosci..

[35] Boehning D., Snyder S.H. (2003). Novel neural modulators. Annu. Rev. Neurosci..

[36] Ungless M.A., Whistler J.L., Malenka R.C., Bonci A. (2001). Single cocaine exposure *in vivo* induces long-term potentiation in dopamine neurons. Nature.

[37] Karler R., Turkanis S.A., Partlow L.M., Calder L.D. (1991). Calcium channel blockers in behavioral sensitization. Life Sci..

[38] Kalivas P.W., Alesdatter J.E. (1993). Involvement of NMDA receptor stimulation in the VTA and amygdala in behavioral sensitization to cocaine. J. Pharmacol. Exp. Ther..

[39] Licata S.C., Schmidt H.D., Pierce R.C. (2004). Suppressing calcium/calmodulin-dependent protein kinase II activity in the ventral tegmental area enhances the acute behavioural response to cocaine but attenuates the initiation of cocaine-induced behavioural sensitization in rats. Eur. J. Neurosci..

[40] Huettner J.E. (1991). Competitive antagonism of glycine at the N-methyl-D-aspartate (NMDA) receptor. Biochem. Pharmacol..

[41] Sola C., Tusell J.M., Serratosa J. (1999). Comparative study of the distribution of calmodulin kinase II and calcineurin in the mouse brain. J. Neurosci. Res..

[42] Nakamura Y., Kitani T., Okuno S., Otake K., Sato F., Fujisawa H. (2000). Immunohistochemical study of the distribution of Ca(2+)/calmodulin-dependent protein kinase phosphatase in the rat central nervous system. Brain Res. Mol. Brain Res..

[43] Dubnau J., Tully T. (1998). Gene discovery in Drosophila: New insights for learning and memory. Annu. Rev. Neurosci..

[44] Ho N., Liauw J.A., Blaeser F., Wei F., Hanissian F., Muglia L.M., Wozniak D.F., Nardi A., Arvin K.L., Holtzman D.M. (2000). Impaired synaptic plasticity and cAMP response element-binding protein activation in Ca2+/calmodulin-dependent protein kinase type IV/Gr-deficient mice. J. Neurosci..

[45] Silva A.J., Paylor R., Wehner J.M., Tonegawa S. (1992). Impaired spatial learning in alpha-calcium-calmodulin kinase II mutant mice. Science.

[46] Matthews R.P., Guthrie C.R., Wailes L.M., Zhao X., Means A.R., Mcknight G.S. (1994). Calcium/calmodulin-dependent protein kinases types II and IV differentially regulate CREB-dependent gene expression. Mol. Cell Biol..

[47] Curtis J., Finkbeiner S. (1999). Sending signals from the synapse to the nucleus: Possible roles for CaMK, Ras/ERK, and SAPK pathways in the regulation of synaptic palsticity and neuronal growth. J. Neurosci. Res..

[48] Lim J., Yang C., Hong S.J., Kim K.S. (2000). Regulation of tyrosine hydroxylase gene transcription by the cAMP-signaling pathway: Involvement of multiple transcription factors. Mol. Cell Biochem..

[49] Onn S.P., Grace A.A. (2000). Amphetamine withdrawal alters bistable states and cellular coupling in rat prefrontal cortex and nucleus accumbens neurons recorded *in vivo*. J. Neurosci..

[50] Robinson T.E., Gorny G., Mitton E., Kolb B. (2001). Cocaine self-administration alters the morphology of dendrites and dendritic spines in the nucleus accumbens and neocortex. Synapse.

[51] Norrholm S.D., Bibb J.A., Nestler E.J., Ouimet C.C., Taylor J.R., Greengard P. (2003). Cocaine-induced proliferation of dendritic spines in nucleus accumbens is dependent on the activity of cyclin-dependent kinase-5. Neuroscience.

[52] Karler R., Finnegan K.T., Clader L.D. (1993). Blockade of behavioral sensitization to cocaine and amphetamine by inhibitors of protein synthesis. Brain Res..

[53] Fujiwara Y., Kazahaya Y., Nakashima M., Sato M., Otsuki S. (1987). Behavioral sensitization to metamphetamine in the rat: An ontogenic study. Psychopharmacology.

[54] Fumagalli F., Bedogni F., Frasca A., di Pasquale L., Racagni G., Riva M.A. (2006). Cortico-striatal up-regulation of activity regulated cytoskeleton-associated protein (Arc) expression following repeated exposure to cocaine. Mol. Pharmacol..

[55] Albertson D.N., Pruetz B., Schmidt C.J., Kuhn D.M., Kapatos G., Bannon M.J. (2004). Gene expression profile of the nucleus accumbens of human cocaine abusers: Evidence for dysregulation of myelin. J. Neurochem..

[56] McClung C.A., Nestler E.J. (2003). Regulation of gene expression and cocaine reward by CREB and detaFosB. Nat. Neurosci..

[57] Robinson T.E., Kolb B. (1999). Alterations in the morphology of dendrites and dendritic spines in the nucleus accumbens and prefrontal cortex following repeated treatment with amphetamine or cocaine. Eur. J. Neurosci..

[58] Grimm J.W., Lu L., Hayashi T., Hope B.T., Su T.P., Shaham Y. (2003). Time-dependent increases in brain-derived neurotrophic factor protein levels within the mesolimbic dopamine system after withdrawal from cocaine: implications for incubation of cocaine craving. J. Neurosci..

[59] Lu L., Grimm J.W., Hope B.T., Shaham Y. (2004). Incubation of cocaine craving after withdrawal: A review of preclinical data. Neuropharmacology.

[60] Bramham C.R., Messaoudi E. (2005). BDNF function in adult synaptic plasticity: The synaptic consolidation hypothesis. Prog. Neurobiol..

[61] Pitchers K.K., Balfour M.E., Lehman M.N., Richtand N.M., Yu L., Coolen L.M. (2010). Neuroplasticity in the mesolimbic system induced by natural reward and subsequent reward abstinence. Biol. Psychiatry.

[62] Hemby S.E., Horman B., Tang W. (2005). Differential regulation of ionotropic glutamate receptor subunits following cocaine self-administration. Brain Res..

[63] Hemby S.E., Co C., Koves T.R., Smith J.E., Dworkin S.I. (1997). Differences in extracellular dopamine concentrations in the nucleus accumbens during response-dependent and response-independent cocaine administration in the rat. Psychopharmacology (Berl.).

[64] You Z.B., Wang B., Zitzman D., Azari S., Wise R.A. (2007). A role for conditioned ventral tegmental glutamate release in cocaine seeking. J. Neurosci..

[65] Chen B.T., Bowers M.S., Martin M., Hopf F.W., Guillory A.M., Carelli R.M., Chou J.K., Bonci A. (2008). Cocaine but not natural reward self-administration nor passive cocaine infusion produces persistent LTP in the VTA. Neuron.

[66] Bird M.K., Reid C.A., Chen F., Tan H.O., Petrou S., Lawrence A.J. (2010). Cocaine-mediated synaptic potentiation is absent in VTA neurons from mGlu5-deficient mice. Int. J. Neuropsychopharmacol..

[67] Kasanetz F., Deroche-Gamonet V., Berson N., Balado E., Lafourcade M., Manzoni O., Piazza P.V. (2010). Transition to addiction is associated with a persistent impairment in synaptic plasticity. Science.

[68] Conrad K.L., Tseng K.Y., Uejima J.L., Reimers J.M., Heng L.J., Shaham Y., Marinelli M., Wolf M.E. (2008). Formation of accumbens GluR2-lacking AMPA receptors mediates incubation of cocaine craving. Nature.

[69] Goldstein R.Z., Volkow N.D. (2002). Drug addiction and its underlying neurobiological basis: Neuroimaging evidence for the involvement of the frontal cortex. Am. J. Psychiatry.

[70] Rilling J., Gutman D., Zeh T., Pagnoni G., Berns G., Kilts C. (2002). A neural basis for social cooperation. Neuron.

[71] Kolb B., Pellis S., Robinson T.E. (2004). Plasticity and functions of the orbital frontal cortex. Brain Cogn..

[72] Volkow N.D., Chang L., Wang G.J., Fowler J.S., Ding Y.S., Sedler M., Logan J., Franceschi D., Gatley J., Hitzemann R. (2001). Low level of brain dopamine D2 receptors in methamphetamine abusers: Association with metabolism in the orbitofrontal cortex. Am. J. Psychiatry.

[73] Kalivas P.W., Volkow N.D. (2005). The neural basis of addiction: A pathology of motivation and choice. Am. J. Psychiatry.

[74] Willeit M., Ginovart N., Graff A., Rusjan P., Vitcu I., Houle S., Seeman P., Wilson A.A., Kapur S. (2008). First human evidence of d-amphetamine induced displacement of a D2/3 agonist radioligand: A [11C]-(+)-PHNO positron emission tomography study. Neuropsychopharmacology.

[75] Boileau I., Dagher A., Leyton M., Gunn R.N., Baker G.B., Diksic M., Benkelfat C. (2006). Modeling sensitization to stimulants in humans: an [11C]raclopride/positron emission tomography study in healthy men. Arch. Gen. Psychiatry.

[76] Volkow N.D., Fowler J.S., Wang G.J., Swanson J.M. (2004). Dopamine in drug abuse and addiction: Results from imaging studies and treatment implications. Mol. Psychiatry.

[77] Volkow N.D., Wang G.J., Ma Y., Fowler J.S., Wong C., Ding Y.S., Hitzemann R., Swanson J.M., Kalivas P. (2005). Activation of orbital and medial prefrontal cortex by methylphenidate in cocaine-addicted subjects but not in controls: Relevance to addiction. J. Neurosci..

[78] Volkow N.D., Wang G.J., Fowler J.S., Thanos P.P., Logan J., Gatley S.J., Gifford A., Ding Y.S., Wong C., Pappas N. (2002). Brain DA D2 receptors predict reinforcing effects of stimulants in humans: Replication study. Synapse.

[79] Volkow N.D. (2004). Imaging the addicted brain: From molecules to behavior. J. Nucl. Med..

[80] Volkow N.D., Wang G.J., Fischman M.W., Foltin R.W., Fowler J.S., Abumrad N.N., Vitkun S., Logan J., Gatley S.J., Pappas N. (1997). Relationship between subjective effects of cocaine and dopamine transporter occupancy. Nature.

[81] Martinez D., Broft A., Foltin R.W., Slifstein M., Hwang D.R., Huang Y., Perez A., Frankle W.G., Cooper T., Kleber H.D. (2004). Cocaine dependence and d2 receptor availability in the functional subdivisions of the striatum: Relationship with cocaine-seeking behavior. Neuropsychopharmacology.

[82] Volkow N.D., Fowler J.S., Wolf A.P., Schlyer D., Shiue C.Y., Alpert R., Dewey S.L., Logan J., Bendriem B., Christman D. (1990). Effects of chronic cocaine abuse on postsynaptic dopamine receptors. Am. J. Psychiatry.

[83] Martinez D., Carpenter K.M., Liu F., Slifstein M., Broft A., Friedman A.C., Kumar D., van Heertum R., Kleber H.D., Nunes E. (2011). Imaging dopamine transmission in cocaine dependence: Link between neurochemistry and response to treatment. Am. J. Psychiatry.

[84] Zalcman S., Savina I., Wise R.A. (1999). Interleukin-6 increases sensitivity to the locomotor-stimulating effects of amphetamine in rats. Brain Res..

[85] Nakajima A., Yamada K., Nagai T., Uchiyama T., Miyamoto Y., Mamiya T., He J., Nitta A., Mizuno M., Tran M.H. (2004). Role of tumor necrosis factor-alpha in methamphetamine-induced drug dependence and neurotoxicity. J. Neurosci..

[86] Maeda T., Kiguchi N., Fukazawa Y., Yamamoto A., Ozaki M., Kishioka S. (2007). Peroxisome proliferator-activated receptor gamma activation relieves expression of behavioral sensitization to methamphetamine in mice. Neuropsychopharmacology.

[87] Narita M., Suzuki M., Kuzumaki N., Miyatake M., Suzuki T. (2008). Implication of activated astrocytes in the development of drug dependence: Differences between methamphetamine and morphine. Ann. N.Y. Acad. Sci..

[88] Xu J., Chavis J.A., Racke M.K., Drew P.D. (2006). Peroxisome proliferator-activated receptor-alpha and retinoid X receptor agonists inhibit inflammatory responses of astrocytes. J. Neuroimmun..

[89] Xu J., Racke M.K., Drew P.D. (2007). Peroxisome proliferator-activated receptor-alpha agonist fenofibrate regulates IL-12 family cytokine expression in the CNS: Relevance to multiple sclerosis. J. Neurochem..

[90] Drew P.D., Xu J., Storer P.D., Chavis J.A., Racke M.K. (2006). Peroxisome proliferator-activated receptor agonist regulation of glial activation: Relevance to CNS inflammatory disorders. Neurochem. Int..

[91] Kubera M., Filip M., Basta-Kaim A., Nowak E., Siwanowicz J., Zajicova A., Holan V., Maes M., Lason W. (2004). The effect of cocaine sensitization on mouse immunoreactivity. Eur. J. Pharmacol..

[92] Irwin M.R., Olmos L., Wang M., Valladares E.M., Motivala S.J., Fong T., Newton T., Butch A., Olmstead R., Cole S.W. (2006). Cocaine dependence and acute cocaine induce decreases of monocyte proinflammatory cytokine expression across the diurnal period: Autonomic mechanisms. J. Pharmacol. Exp. Ther..

[93] Schmidt A., Vogel R., Holloway M.K., Rutledge S.J., Friedman O., Yang Z., Rodan G.A., Friedman E. (1999). Transcription control and neuronal differentiation by agents that activate the LXR nuclear receptor family. Mol. Cell. Endocrinol..

[94] Delerive P., Fruchart J.C., Staels B. (2001). Peroxisome proliferator-activated receptors in inflammation control. J. Endocrinol..

[95] Lleo A., Galea E., Sastre M. (2007). Molecular targets of non-steroidal anti-inflammatory drugs in neurodegenerative diseases. Cell Mol. Life Sci..

[96] Peters J.M., Hennuyer N., Staels B., Fruchart J.C., Fievet C., Gonzalez F.J., Auwerx J. (1997). Alterations in lipoprotein metabolism in peroxisome proliferator-activated receptor alpha-deficient mice. J. Biol. Chem..

[97] Akiyama T.E., Nicol C.J., Fievet C., Staels B., Ward J.M., Auwerx J., Lee S.S., Gonzalez F.J., Peters J.M. (2001). Peroxisome proliferator-activated receptor-alpha regulates lipid homeostasis, but is not associated with obesity: Studies with congenic mouse lines. J. Biol. Chem..

[98] Cuzzocrea S., Mazzon E., di Paola R., Peli A., Bonato A., Britti D., Genovese T., Muià C., Crisafulli C., Caputi A.P. (2006). The role of the peroxisome proliferator-activated receptor-alpha (PPAR-alpha) in the regulation of acute inflammation. J. Leukoc. Biol..

[99] Fernandez-Espejo E., Ramiro-Fuentes S., Rodriguez de Fonseca F. (2009). The absence of a functional peroxisome proliferator-activated receptor-alpha gene in mice enhances motor sensitizing effects of morphine, but not cocaine. Neuroscience.

[100] Melis M., Pillolla G., Luchicchi A., Montoni A.L., Yasar S., Goldberg S.R., Pistis M. (2008). Endogenous fatty acid ethanolamides supress nicotine-induced activation of mesolimbic dopamine neurons through nuclear receptors. J. Neurosci..

[101] Berhow M.T., Russell D.S., Terwilliger R.Z., Beitner-Johnson D., Self D.W., Lindsay R.M., Nestler E.J. (1995). Influence of neurotrophic factors on morphine- and cocaine-induced biochemical changes in the mesolimbic dopamine system. Neuroscience.

[102] Berhow M.T., Hiroi N., Nestler E.J. (1996). Regulation of ERK extracellular signal regulated kinase, part of the neurotrophin signal transduction cascade, in the rat mesolimbic dopamine system by chronic exposure to morphine or cocaine. J. Neurosci..

[103] Sklair-Tavron L., Shi W.X., Lane S.B., Harris H.W., Bunney B.S., Nestler E.J. (1996). Chronic morphine induces visible changes in the morphology of mesolimbic dopamine neurons. Proc. Nat. Acad. Sci. USA.

[104] Messer C.J., Eisch A.J., Carlezon W.A., Whisler K., Shen L., Wolf D.H., Westphal H., Collins F., Russell D.S., Nestler E.J. (2000). Role for GDNF in biochemical and behavioral adaptations to drugs of abuse. Neuron.

[105] Flores C., Rodaros D., Stewart J. (1998). Long-lasting induction of astrocytic basic fibroblast growth factor by repeated injections of amphetamine: Blockade by concurrent treatment with a glutamate antagonist. J. Neurosci..

[106] Flores C., Stewart J. (2000). Basic fibroblast growth factor as a mediator of the effects of glutamate in the development of long-lasting sensitization to stimulant drugs: Studies in the rat. Psychopharmacology.

[107] Reuss B., von Bohlen und Halbach O. (2003). Fibroblast growth factors and their receptors in the central nervous system. Cell Tissue Res..

[108] Flores J.A., Galan-Rodriguez B., Rojo A.I., Ramiro-Fuentes S., Cuadrado A., Fernandez-Espejo E. (2010). Fibroblast growth factor-1 within the ventral tegmental area participates in motor sensitizing effects of morphine. Neuroscience.

[109] Fumagalli F., di Pasquale L., Caffino L., Racagni G., Riva M.A. (2008). Stress and cocaine interact to modulate basic fibroblast growth factor (FGF-2) expression in rat brain. Psychopharmacology (Berl.).

[110] Wang C.Y., Yang F., He X., Chow A., Du J., Russell J.T., Lu B. (2001). Ca(2+) binding protein frequenin mediates GDNF-induced potentiation of Ca(2+) channels and transmitter release. Neuron.

[111] Grimm J.W., Lu L., Hayashi T., Hope B.T., Su T.P., Shaham Y. (2003). Time-dependent increases in brain-derived neurotrophic factor protein levels within the mesolimbic dopamine system after withdrawal from cocaine: Implications for incubation of cocaine craving. J. Neurosci..

[112] Le Foll B., Diaz J., Sokoloff P. (2005). A single cocaine exposure increases BDNF and D3 receptor expression: Implications for drug-conditioning. Neuroreport.

[113] Graham D.L., Edwards S., Bachtell R.K., di Leone R.J., Rios M., Self D.W. (2007). Dynamic BDNF activity in nucleus accumbens with cocaine use increases self-administration and relapse. Nat. Neurosci..

[114] Russo S.J., Mazei-Robison M.S., Ables J.L., Nestler E.J. (2009). Neurotrophic factors and structural plasticity in addiction. Neuropharmacology.

[115] Berglind W.J., See R.E., Fuchs R.A., Ghee S.M., Whitfield T.W., Miller S.W., McGinty J.F. (2007). A BDNF infusion into the medial prefrontal cortex suppresses cocaine seeking in rats. Eur. J. Neurosci..

[116] Lu H., Cheng P.L., Lim B.K., Khoshnevisrad N., Poo M.M. (2010). Elevated BDNF after cocaine withdrawal facilitates LTP in medial prefrontal cortex by suppressing GABA inhibition. Neuron.

[117] Fanous S., Lacagnina M.J., Nikulina E.M., Hammer R.P. (2011). Sensitized activation of Fos and brain-derived neurotrophic factor in the medial prefrontal cortex and ventral tegmental area accompanies behavioral sensitization to amphetamine. Neuropharmacology.

[118] Finkbeiner S., Tavazoie S.F., Maloratsky A., Jacobs K.M., Harris K.M., Greenberg M.E. (1997). CREB: A major mediator of neuronal neurotrophin responses. Neuron.

[119] Tao X., Finkbeiner S., Arnold D.B., Shaywitz A.J., Greenberg M.E. (1998). Ca2+ influx regulates BDNF transcription by a CREB family transcription factor-dependent mechanism. Neuron.

